# Hemodynamics in femoro-femoral venovenous extracorporeal membrane oxygenation using large eddy simulations

**DOI:** 10.1038/s41598-025-22403-6

**Published:** 2025-10-09

**Authors:** Hanna Hörwing, Louis Parker, Anders Svensson-Marcial, Torkel B. Brismar, Lars Mikael Broman, Lisa Prahl Wittberg

**Affiliations:** 1 Department of Engineering Mechanics, FLOW, KTH Royal Institute of Technology, Stockholm, Sweden; 2 Laboratoire d’Imagerie Biomédicale (LIB), Sorbonne Université, Paris, France; 3 ICAN Imaging, Institute of Cardiometabolism and Nutrition (ICAN), Paris, France; 4 School of Engineering, University of Western Australia, Perth, Australia; 5 Department of Radiology, Karolinska University Hospital, Stockholm, Sweden; 6 Unit of Radiology, Department of Clinical Science, Intervention and Technology, Karolinska Institute, Stockholm, Sweden; 7 ECMO Centre Karolinska, Pediatric Perioperative Medicine and Intensive Care, Karolinska University Hospital, Stockholm, Sweden; 8 Department of Physiology and Pharmacology, Karolinska Institute, Stockholm, Sweden

**Keywords:** Venovenous, Extracorporeal membrane oxygenation, Femoro-femoral cannulation, Recirculation, Wall shear stress, Computational fluid dynamics, Biomedical engineering, Blood flow

## Abstract

Venovenous extracorporeal membrane oxygenation (ECMO) is used for support in refractory severe respiratory failure. Venous drainage and return are accomplished through cannulation of patient’s major veins, typically on the neck and/or the groins. Cannulation configuration may affect treatment efficiency, but it remains unclear if any strategy is superior. Computational fluid dynamics was used to evaluate and compare the femoro-femoral (FF), jugulo-femoral (JF), and femoro-jugular (FJ) cannulation configurations. Cannulae were modelled in an adult patient-averaged geometry of the right atrium and venae cavae. Large eddy simulations were performed at ECMO flow rates of 2–6 L/min. Time-averaged flow data was collected for assessment of flow parameters associated with clinical efficiency. FF cannulation showed lower recirculation than FJ and JF. Negative pressures in the inferior vena cava, associated with an increased risk of vascular collapse, were more pronounced in the FF configuration. Additionally, wall shear stresses exceeded physiological levels even at low flow rates and increased with higher flow, increasing the risk of blood trauma. Shear stress varied significantly inside the drainage cannula, highlighting sensitivity to local flow dynamics. This study advances our understanding of three common VV ECMO configurations, giving insights to improve efficiency and address clinical challenges.

## Introduction

Extracorporeal membrane oxygenation (ECMO) is a life-saving support for critically ill patients with refractory respiratory and/or cardiac failure. The treatment requires blood to be drained from the patient for oxygenation in an extracorporeal circuit, whereafter the blood is returned to the patient. The complete ECMO circuit consists of drainage and return cannulae, a centrifugal pump, a membrane lung (gas exchanger), tubing, and connectors. Oxygenated blood can be returned to the venous or arterial side of the patient’s circulation. When both drainage and return of blood occurs in the venous compartment, the patient is provided with respiratory support. This mode is referred to as venovenous (VV) ECMO. Conversely, when the oxygenated blood is returned to the arterial side, cardio-respiratory support is offered, i.e. venoarterial ECMO.

ECMO mode and cannulation configuration are determined by the specific needs of the patient, along with the availability and preferences of the medical center. For VV ECMO, the jugular and femoral veins are common sites for cannula insertion. In femoro-femoral (FF) cannulation, both the drainage cannula and return cannula are inserted into each respective common femoral vein, with the tips reaching up into the inferior vena cava (IVC) or right atrium (RA). With an FF configuration, the need for cannulation of a vessel in the upper body is eliminated. The use of FF cannulation has been motivated by the relatively easy and rapid access to the femoral veins, at the same time controlling bleeding at the cannulation site with local pressure^1^. In femoro-jugular (FJ) and jugulo-femoral (JF) cannulation, one cannula is instead inserted into the internal jugular vein, with the tip reaching into the superior vena cava (SVC) or into the upper part of the RA. VV ECMO with FF cannulation is referred to as V$_f$-V$_f$ ECMO, where the letters before the hyphen refer to the drainage cannula and those after the hyphen refer to the return cannula. The division using a hyphen marks the membrane lung, and subscripts denote the cannula insertion site^2^. At this level of the nomenclature the placement of the cannula tip, for example, or cannula sizes are not delineated.

From a treatment perspective, as blood is returned in close proximity to the drainage site, a fraction of the oxygenated blood may be drained out back into the ECMO circuit before passing through the tricuspid valve contributing to the patient’s oxygenation. This proportion of ECMO blood drained out is referred to as the recirculation fraction ($R_f$), which consequently decreases the effective ECMO flow. Cannula configuration, cannula positioning, ECMO flow rate, and cardiac output all impact $R_f$^1,3–8^. Parker et al. (2022a) studied the effect of occluded side holes of the drainage cannula on $R_f$, finding that occlusion of side holes increased $R_f$ significantly. Minimizing $R_f$ is key for efficient ECMO support by reducing the flow necessary to maintain adequate oxygenation. Comparative clinical studies on optimal cannulation strategies to minimize recirculation are sparse^6^. Nonetheless, FF has been associated with greater recirculation than the more common FJ^9–11^. Alternatively, dual-lumen cannula cannulation has been seen to further minimize recirculation in VV ECMO compared to single lumen cannulation^4,12^.

For blood, non-Newtonian shear thinning behaviour^13^ is primarily observed at low shear rates, while at higher shear rates ($>100$ /s), the viscosity becomes nearly constant^14^. Wyk et al. (2013)^15^ evaluated wall shear stress (WSS) variations near bifurcations for a non-Newtonian, blood-like fluid. With separation and secondary flows developing in these bifurcations, the authors highlighted the importance of incorporating shear-thinning non-Newtonian viscosity parameters when evaluating physiological flow features. Non-physiological flow conditions, originating from the interaction of the fluid with the ECMO circuit and surrounding vessels, give rise to flow separation, areas of low and oscillatory shear stress, as well as increased residence times^16,17^. These altered hemodynamic conditions contribute to an enhanced risk of platelet activation and hemolysis^16,18–22^. Vessel occlusion generates high shear stresses as the blockage requires higher blood velocity to sustain the same mass flow rate^16,17^. Unphysiologically high shear can potentially induce rupture of red blood cells (hemolysis), and activate platelets, inducing thrombosis. Further, von Willebrand factor (vWF), a protein essential for clot formation, is particularly reactive to high shear stresses^22–24^. Under the described conditions, the protein may unfold and become adhesive, enabling platelet aggregation. In contrast, lower stress levels below the level at which red blood cells rupture, may still activate platelets increasing the risk of thrombus formation^19^. Low shear stresses occur in stagnant regions, where residence times are high. Thresholds for different types of blood trauma have been found, determined by shear stress and exposure time^18^. To avoid blood trauma entirely in medical devices, Chan et al. (2022) stated that shear stress should be kept below 12 Pa.

Introducing cannulae into vessels may further give rise to non-physiological pressures and pressure variations^25,26^. A review study by Patel et al. (2019) stated that excessive negative pressures at the drainage cannula could cause the vena cava to collapse around the cannula, and hence obstruct the (drainage) side holes. In the clinic, this may in turn lead to typical vibrations of the tubing and cannula, termed “chattering”. Drainage insufficiency with frequent line chattering is associated with elevated $R_f$, hemolysis and potential damage of the venous vessel walls^27,28^.

Given the practical limitations of conducting in vivo studies on cannulation techniques, computational fluid dynamics (CFD) simulations have been used to study the flow through cannulae in ideal geometries^29,30^; dual-lumen cannula configuration^4^; as well as JF and FJ configurations^3^. Numerical research remains scarce regarding FF cannulation and further insights are needed to establish comparative data for cannulation strategies. Direct comparisons between different cannulation strategies are lacking, and the choice of cannulation strategy often lands on the preferences of the medical center.

This work numerically investigated blood flow dynamics in FF cannulation, with regards to pressures at drainage site, stagnation of blood, $R_f$, and mechanical stresses. Clinical risks associated with various flow phenomena were evaluated for different cannula configurations and flow conditions. The aim was to increase knowledge for clinicians to make informed decisions. We hypothesize that the cannulation configuration governs atrial flow dynamics, affecting circuit efficiency, and the risk of clinical complications related to blood trauma.

## Methods

### Study population and 3D model reconstruction

**Fig. 1:**
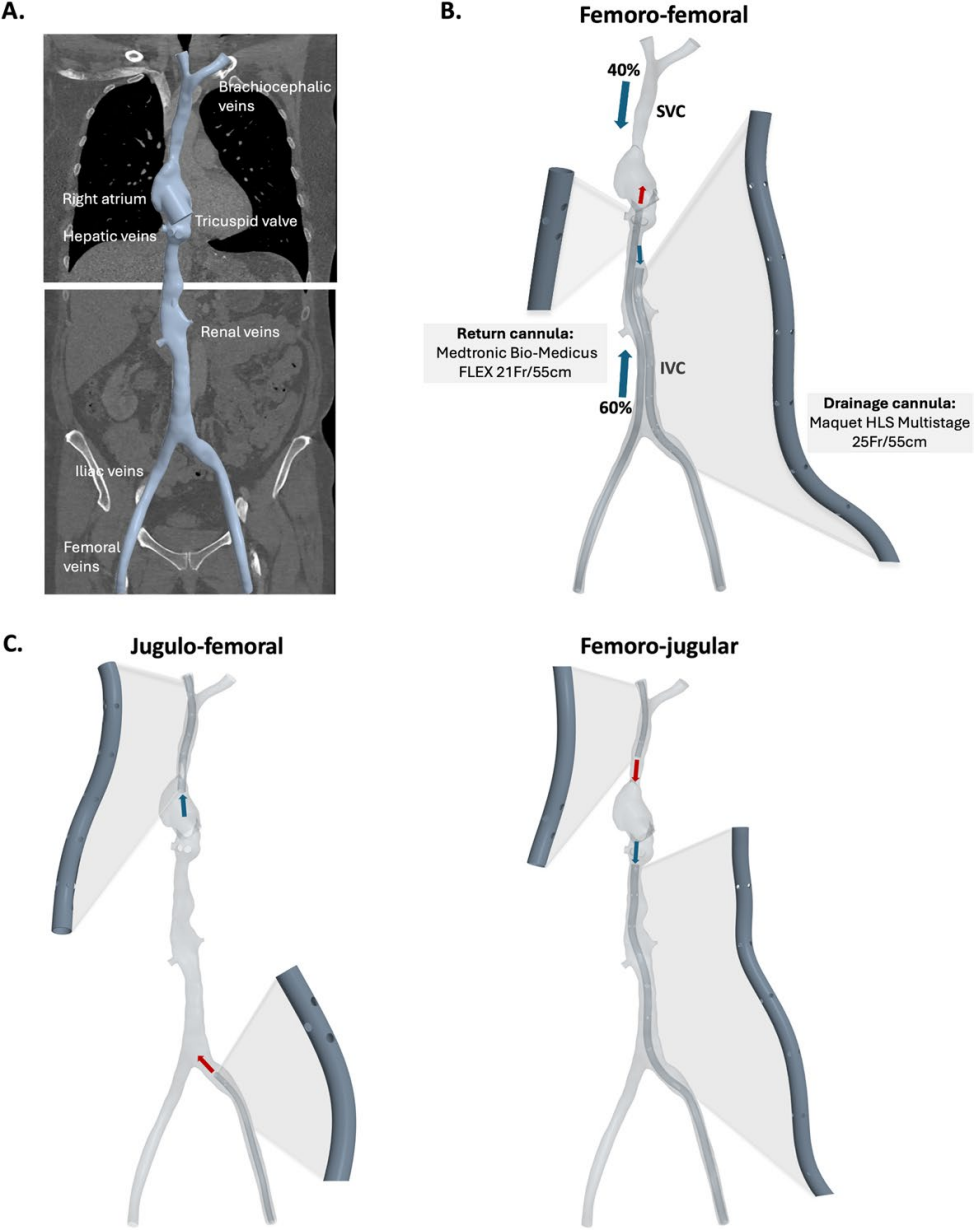
**A.** Computed tomography scan and patient-averaged 3D reconstruction of the right atrium and main venous branches. **B.** Cannulae in femoro-femoral configuration inserted into 3D reconstruction of the right atrium, inferior vena cava (IVC) and superior vena cava (SVC). **C.** Cannulae in jugulo-femoral and femoro-jugular configurations, inserted into 3D reconstruction.

A previously published patient-averaged model^31^ of the IVC, SVC, RA and major connecting veins was used for the study and extended to also include femoral and hepatic veins. The patient-averaged model was based on four unique patients’ computed tomography (CT) venograms. Two CT acquisitions were performed in each of four healthy subjects (3 female, 1 male; age $58.3 \pm 3.5$ yrs; weight $77.0 \pm 11.9$ kg), one of the upper venous system and one of the lower venous system. The averaging was performed by scaling the dimensions of the venous branches and the RA to create a single representative model, capturing the generalized anatomy shown in Fig. 1A.

**Table 1:** Specification for drainage and return cannulae used in the study for femoro-femoral (FF), femoro-jugular (FJ), and jugulo-femoral (JF) configurations. 1 Fr = 1/3 mm.

Cannula	Size	Type	Side holes	Configuration
	Outer diameter / Max. insertion length		Rows / Holes per row / Diameter	
Drainage	25 Fr / 55 cm	Multistage	6 / 4 / 2.5 mm	FF, FJ
Drainage	25 Fr / 38 cm	Multistage	5 / 4 / 2.5 mm	JF
Return	21 Fr / 55 cm	Lighthouse	3 / 2 / 2.6 mm	FF
Return	19 Fr / 18 cm	Lighthouse	3 / 2 / 2.6 mm	FJ, JF

The cannulae used in this study are listed in Table 1. For the JF and FJ configurations, cannulae are identical to those studied in Parker et al. (2022a). For the FF configuration, cannulae are based on the geometry of a Maquet/Getinge HLS 25Fr/55cm (Rastatt, Germany) as the drainage cannula and a Medtronic Bio-Medicus FLEX 21Fr/55cm (Tolochenaz, Switzerland) as the return cannula. These were created in STAR-CCM+ (Siemens, Munich) computer-aided design (CAD) client and placed in the rigid-wall geometry described above (Fig. 1B), following the anatomy of the vessel. For jugular drainage (JF), the tip of the drainage cannula was placed approximately 1.5–2 cm into the RA (Fig. 1C). In the FJ configuration, the femoral drainage cannula was positioned distal to the junction of the hepatic veins. In the FF configuration, the femoral return was positioned at the base of the RA, with the drainage tip positioned 6 cm distal to the return cannula tip.

The model used by Parker et al. (2022a) did not account for the hepatic veins, which contribute approximately 25% of the blood inflow to the RA. To compare cannula performance across different configurations, this limitation was addressed by re-simulating JF and FJ with a model that incorporated hepatic veins. Simulations were also performed on the new geometry with no cannulae present.

### Governing equations

In fluid dynamics, the motion of a fluid is described by the governing equations, expressing fundamental conservation principles. These equations form the basis for modelling the motion of fluids, such as blood flows. The conservation of mass is expressed through the continuity equation as follows:

1 $$\begin{aligned} \frac{\partial \rho }{\partial t} + \nabla \cdot (\rho \textbf{u}) = 0 \end{aligned}$$

with $\rho$ being the density and $\textbf{u}$ the velocity field. For incompressible flow, the continuity equation is reduced to $\nabla \cdot \textbf{u} = 0$, enforcing zero divergence of the velocity field. Moreover, the conservation of momentum is expressed by the Navier-Stokes equation:

2 $$\begin{aligned} \rho \left( \frac{\partial \textbf{u}}{\partial t} + (\textbf{u} \cdot \nabla )\textbf{u} \right) = \nabla \cdot \varvec{\sigma } + \textbf{f} \end{aligned}$$

where $\varvec{\sigma }$ is the stress tensor and $\textbf{f}$ represents additional body forces. For incompressible flows, the stress tensor can be modelled as a function of the hydrostatic pressure, $p$, dynamic viscosity, $\mu$, and shear rate tensor, $\dot{\gamma }$:

3 $$\begin{aligned} \varvec{\sigma } = -p \textbf{I} + 2 \mu \dot{\gamma } \end{aligned}$$

The dynamic viscosity is non-constant in non-Newtonian fluids such as blood. Modelling of the viscosity will be further explained in the next section.

### Computational model and hemodynamics

Simulations on the 3D model were performed in STAR-CCM+ (v.18.06.007). The initial flow fields were obtained using steady Reynolds-averaged Navier-Stokes (RANS) k-$\omega$ SST model with a segregated solver. The SIMPLE algorithm was used for pressure–velocity coupling, and convective terms were discretized with a second-order upwind scheme. Convergence was monitored by reducing the residuals below $10^{-5}$, while key parameters, including velocity, pressure and $R_f$, were tracked to ensure they reached a converged state. After obtaining a steady flow field, large eddy simulations (LES) with a wall-adapting local eddy-viscosity (WALE) subgrid-scale (SGS) model using a bounded-central difference convection scheme were initiated with second-order temporal discretization.

The time step was set to be constant at $10^{-4}$ s and simulations were run for 3 s physical time at ECMO flow rates of 2–6 L/min. Constant flow rates were prescribed at the inlets, with 60% of the cardiac output (CO) returning to the RA via the IVC (25% from hepatic veins; 20% from renal veins; 15% from the iliac veins), 38% returning via the SVC and 2% via the coronary sinus. The CO was set to 6 L/min for all cases and blood density was 1050 kg/m${^3}$. Walls were assumed to be rigid and a no-slip condition was applied. The tricuspid valve was modelled as a zero-pressure outlet. The computational model followed the same methodology as previous studies on cannulae and hemodynamics^3,31,32^.

A grid convergence study was conducted on three different meshes comparing $R_f$, turbulent kinetic energy (TKE) and velocity profiles with RMS along different probe lines throughout the geometry. The resulting core mesh was made up of 10.4M polyhedral cells with 6 prism layers at the boundary walls. The meshing parameters used have previously been determined to be sufficiently resolved for LES simulations of the RA^31^. Inlets and outlets were extruded with 10-times vessel diameter. This was seen to be a sufficient entrance length for all the inlet flow profiles to fully develop, and to avoid any interference with the flow field in the model. The grid sensitivity assessment is provided in Supplemental Material (Table S1, Figure S1 and Figure S2).

Sensitivity to several modelling assumptions has been evaluated in previously published studies^31,32^. Parker et al. (2022c) compared URANS, implicit LES with two convection schemes (second-order upwind and bounded-central difference), and LES with a WALE SGS model, finding the latter to be well suited for characterizing flows in the RA. Further, Parker et al. (2022b) investigated sensitivities with regards to constant/pulsatile inlet flow conditions and hematocrit, finding pulsatile inlet flows to have a negligible impact on time-averaged flow data.

Due to the complexity of the model in this study, shear rates varied highly throughout the domain, making it necessary to incorporate a non-Newtonian viscosity model^13,15^. A Quemada (Cokelet) viscosity model^33,34^ was thereby employed to capture the fluid dynamics at a broad range of shear rates:

4 $$\begin{aligned} \mu =\mu _P\left[ 1-\frac{\alpha }{2} \frac{k_0+k_{\infty } \sqrt{\dot{\gamma } / \dot{\gamma }_c}}{1+k_{\infty } \sqrt{\dot{\gamma } / \dot{\gamma }_c}}\right] ^{-2} \end{aligned}$$

where $\mu _P$ corresponds to the plasma viscosity, $\alpha$ the hematocrit level and $\dot{\gamma }$ the scalar form of the shear rate. Based on previous studies^31,32^, hematocrit was set to 35%. Model parameters $k_0$, $k_{\infty }$ and $\dot{\gamma }_c$ have been fitted to experiments by Cokelet et al. (2005):

5 $$\begin{aligned} k_0=\exp \left( 3.874-10.41 \alpha +13.8 \alpha ^2-6.738 \alpha ^3\right) \end{aligned}$$

6 $$\begin{aligned} k_{\infty }=\exp \left( 1.3435-2.803 \alpha +2.711 \alpha ^2-0.6479 \alpha ^3\right) \end{aligned}$$

7 $$\begin{aligned} \dot{\gamma }_c=\exp \left( -6.1508+27.923 \alpha -25.6 \alpha ^2+3.697 \alpha ^3\right) \textrm{s}^{-1} \end{aligned}$$

Oscillatory shear index (OSI) is a dimensionless parameter used to evaluate wall shear stress oscillations by quantifying the changes in direction and magnitude of WSS. $\text {OSI} = 0$ corresponds to a unidirectional flow and $\text {OSI} = 0.5$ corresponds to a purely oscillatory flow. Time-averaged wall shear stress (TAWSS) and OSI were defined as follows:

8 $$\begin{aligned} \text {TAWSS} = \frac{1}{T} \int _{0}^{T} \vert \mathbf {\tau }_w (t) \vert dt, \end{aligned}$$

9 $$\begin{aligned} OSI = \frac{1}{2} \left( 1 - \frac{\left| \int _{0}^{T} \vec {\tau }_w (t) dt\right| }{\int _{0}^{T} \left| \vec {\tau }_w (t) \right| dt} \right) . \end{aligned}$$

TAWSS, velocity and pressure were computed by averaging over the last second of simulation (Supplemental Material, Figure S3), with data collected every 0.01 s. Stagnation was defined as regions with time-averaged velocity magnitude $<0.001$ m/s. To avoid overestimation of stagnant volume due to the no-slip condition, a threshold volume was created excluding the first 0.1 mm closest to the wall. To study the distribution of oxygenated blood from the return cannula, a passive scalar was introduced at the return cannula inlet. $R_f$ was then defined as:

10 $$\begin{aligned} R_f = \frac{\text {Oxygenated blood from return cannula drained}}{\text {Total blood drained}} \end{aligned}$$

The Reynold’s number for the return jet, based on cannula inner diameter, averaged velocity at the cannula outlet and apparent viscosity, were 2127, 4254 and 6381 for 2, 4, and 6 L/min ECMO flow rates, respectively.

## Results and discussion

### Recirculation fraction, $R_f$

**Fig. 2:**
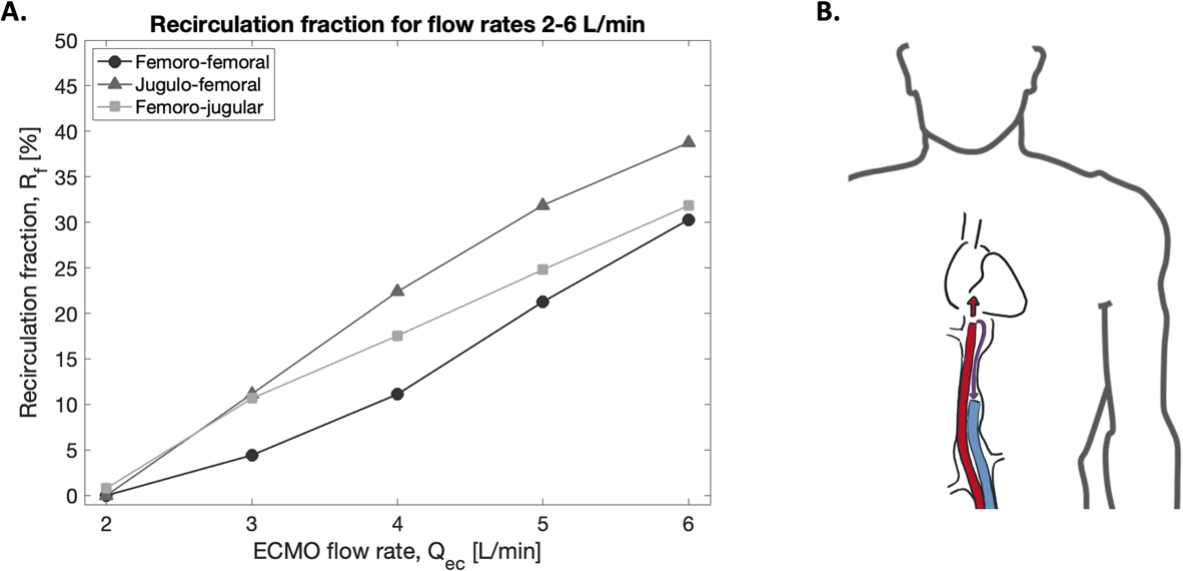
**A.** Recirculation fraction, $R_f$, under femoro-femoral (FF) cannulation in comparison to jugulo-femoral (JF) and femoro-jugular (FJ) configurations. **B.** Diagram depicting the concept of recirculation. Oxygenated blood exiting the return cannula goes into the drainage cannula before passing through the tricuspid valve.

Figure 2B illustrates recirculation, where a portion of the oxygenated blood exiting the return cannula is drawn back into the ECMO circuit via the drainage cannula. Figure 2A shows the fraction of oxygenated (recirculated) blood being drained at various ECMO flow rates. An increase in $R_f$ was observed with increasing flow rates for all three cannula configurations, with values approaching zero at 2 L/min. $R_f$ was lowest for FF across all ECMO flow rates, with a mean difference of 7.4 ± 4.0% compared to JF and 3.7 ± 2.3% compared to FJ. Interestingly, JF attained a notable increase in $R_f$ after hepatic veins were incorporated. This is further compared in Supplemental Material (Figure S5). $R_f$ in FJ and JF configurations was further compared to clinical data measured by Fisser et al. (2022)^8^ and was seen to fall within the 95% confidence interval of the clinical data in each configuration (Supplemental Material, Figure S7).

Previous studies have shown that repositioning of multistage drainage cannulae results in only minor improvements in $R_f$, as the majority of blood in multistage cannulae is drained from the most proximal side holes, where blood is deoxygenated^7^. Moreover, in the JF configuration, earlier CFD results have indicated minimal impact of cannula positioning on $R_f$^3^. After exiting the return cannula, oxygenated blood will according to basic fluid mechanical cannula studies be fully mixed with the native flow after 10-12 cannula diameters, depending on cannula volume flow^35^. From this perspective, keeping the cannulas sufficiently separated should minimize recirculation. With FF cannulation, it has been stated that maintaining cannula tip separation $\ge 8$ cm minimizes $R_f$^1^.

**Fig. 3:**
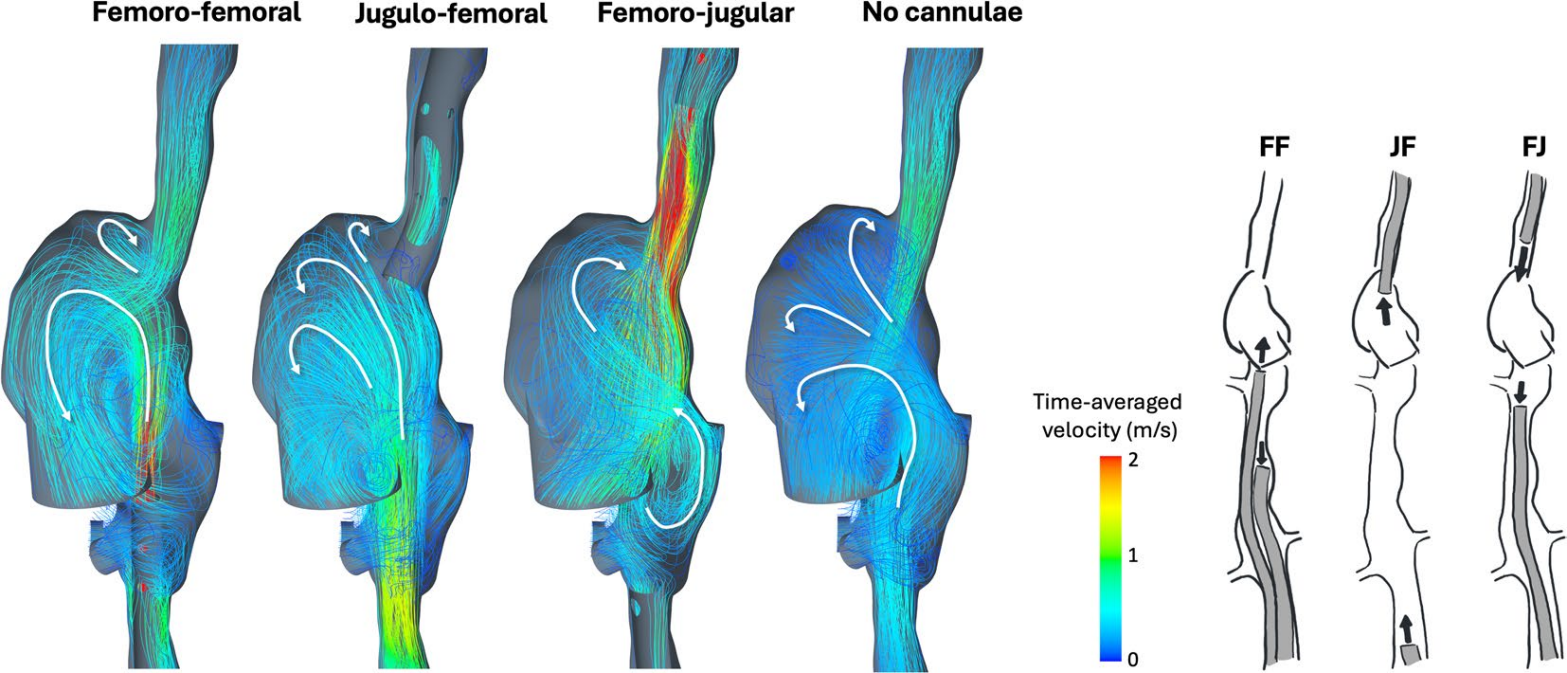
Streamlines of time-averaged velocities in the right atrium for the three cannula configurations femoro-femoral (FF), jugulo-femoral (JF) and femoro-jugular (FJ) compared to case with no cannulae, modified from Parker et al. (2022a).

### Flow structures

Flow structures in the RA under FJ, JF and FF configurations are depicted in Fig. 3. These are shown along with a model with no cannulation for comparison with the native flow. For FF, the flow exiting the return cannula collided with the flow from the SVC, forming a large rotating vortex in the center of the RA. This vortex directed the flow towards the tricuspid valve, maintaining the native rotating structures seen when no cannula was present in the model. With no cannulae, the colliding flows generated helical structures, moving the flow towards the tricuspid valve. This helicity was also present during JF cannulation, but was less apparent in FF. In the FJ configuration, multiple vortices of varying orientation appeared, disrupting the native flow while aiming the flow away from the tricuspid valve.

**Fig. 4:**
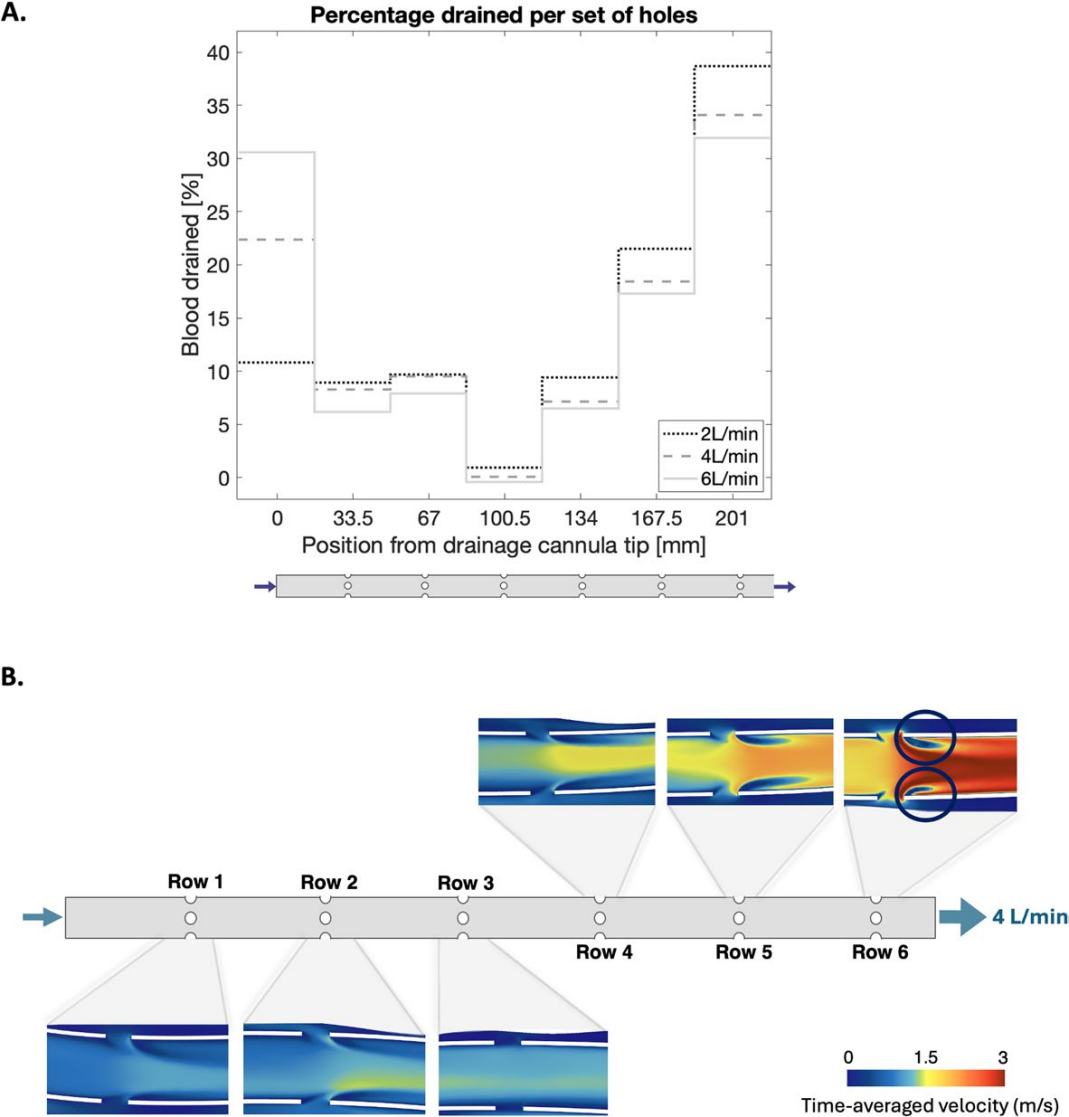
Drainage distribution (%) along the length of the drainage cannula for ECMO flow rates 2, 4 and 6 L/min. Time-averaged velocity in drainage cannula for ECMO flow rate of 4 L/min. Circles highlight separation bubbles at the most proximal side holes, forming downstream of the side holes.

To assess how blood drainage was distributed throughout the cannula during FF cannulation, the drainage fraction was computed at each set of cannula side holes across different ECMO flow rates (Fig. 4A). At low flow rates (2 L/min), almost 40% of the blood was drained at the most proximal side holes in relation to the ECMO pump, while only about 11% was drained from the cannula tip. As flow rate increased, the total drainage flow exceeded that supplied by the femoral and renal veins, leading to an increased proportion of blood being sourced from the region closer to the RA. Thus, at 6 L/min, over 30% of the blood was drained at the tip of the cannula positioned in proximity to the well-oxygenated blood entering via the return cannula, resulting in increased $R_f$.

Retrospective studies have reported comparable clinical outcomes with regards to oxygenation for FF and FJ^36,37^. A multicentre study of 335 patients found arterial oxygen levels to be comparable between the FF and FJ groups, however ECMO-related complications were more frequently encountered with the FJ configuration^36^. Guervilly et al. (2014) found that, with sufficient tip separation and adequate blood flow, the performance of the circuit may not be affected by cannula configuration. However, our results show that as ECMO flow increased, the total drainage flow exceeded the available venous inflow from the femoral and renal veins. This shift caused a greater proportion of the drainage to occur near the right atrium, close to the oxygenated blood exiting the return cannula. At 6 L/min, more than 30% of the blood was drained at the tip, increasing the likelihood of capturing return flow and thereby raising the recirculation fraction. These findings suggest that increasing ECMO flow alters drainage distribution along the cannula, shifting suction toward the tip. Recirculation is thus not solely determined by cannulation configuration but is highly sensitive to the balance between drainage demand and venous supply. Moreover, Palmer et al. (2016) studied the impact of single- and multistage cannulae on $R_f$, finding that the single stage cannula was more affected by positioning, whereas $R_f$ was less sensitive to repositioning of the multistage cannula. Thus, it should be noted that $R_f$ is affected not only by tip distance, but also by factors such as cannula design, flow rates and vessel anatomy.

At row 3 of the cannula side holes, drainage was near-zero. This may simply be due to the presence of two cannulae in the IVC, partly obstructing the flow and limiting the drainage in this region. The same drainage cannula has been studied in VA mode with femoral drainage^38^. Without the obstruction of a second cannula in the IVC, the authors observed an increase in drainage volume from the first row of side holes to the most proximal one. This suggests that the low drainage at row 3 could primarily be a result of spatial obstruction. The drainage cannula was rotated 45$^{\circ }$ to investigate sensitivity to cannula direction, showing no considerable difference in drainage distribution (Supplemental Material, Figure S4).

**Fig. 5:**
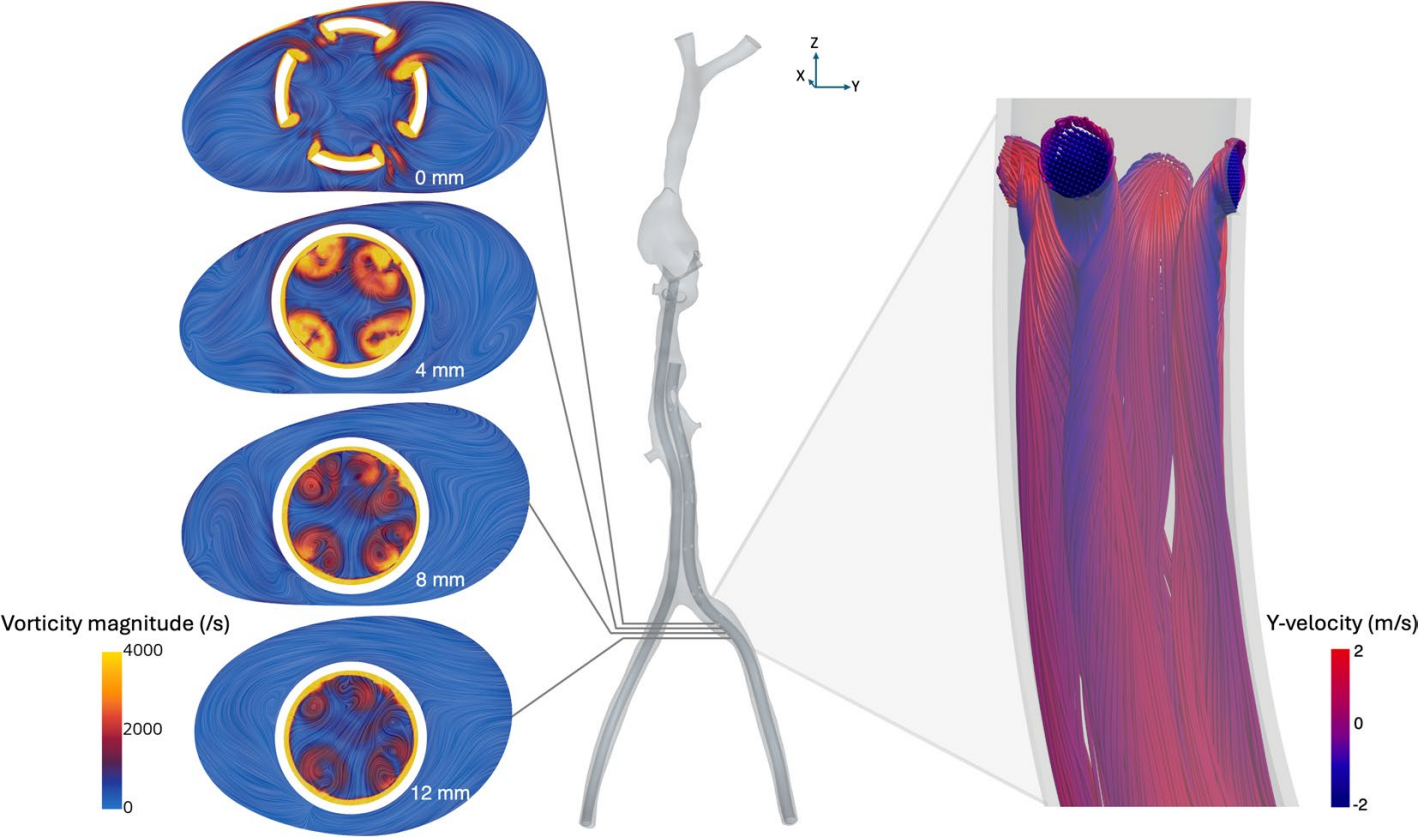
Vorticity magnitude and flow direction in four cross-sectional planes downstream of the most proximal side holes in the drainage cannula. Streamlines entering the drainage cannula at the side holes show the formation of the counter-rotating vortex pairs.

Figure 4B depicts the flow in the drainage cannula at an ECMO flow rate of 4 L/min. Blood enters the drainage cannula through the side holes and collides with the upstream flow within the cannula, generating localized jets in crossflow at each side hole^39^. These jets are most pronounced at the most proximal side holes in relation to the ECMO pump, where flow velocity and drainage are highest. The interaction between the jets and the crossflow generates complex flow structures, including shear layers and counter-rotating vortex pairs (CVPs)^39^ (Fig. 5). The crossflow causes shear layer vorticity to tilt and fold, leading to the formation of the CVP. These vortex pairs influence the flow dynamics by enhancing the entrainment of blood and by acting as a trapping mechanism, temporarily retaining blood within their structures before dissipating approximately 15 mm downstream of the side holes.

**Fig. 6:**
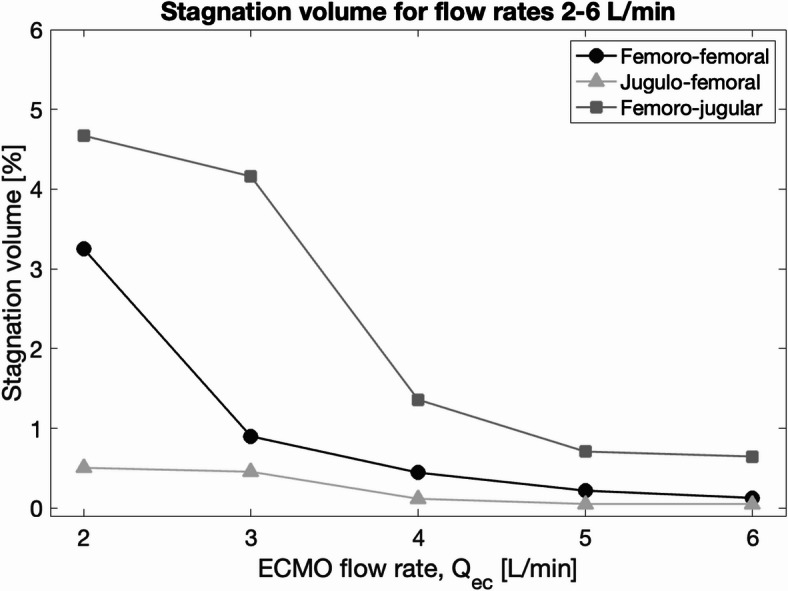
Volume fraction of stagnant blood in femoro-femoral, femoro-jugular and jugulo-femoral configurations.

While entrainment enforces mixing, it also contributes to the formation of stagnation regions downstream of the side holes, marked as circles in Fig. 4B. These regions are often referred to as recirculation zones^29^, but to separate from the clinical term “recirculation”, they will hereafter be referred to as separation bubbles. The presence of separation bubbles increases residence time, promoting stagnation and potential thrombus formation. Similar flow structures have previously been observed in drainage cannulae in ideal geometries^29^, both numerically using CFD and experimentally using particle image velocimetry (PIV). Separation bubbles have further been identified in lighthouse and multistage cannulae under FJ configurations^40^ and in multistage cannulae operating in VA mode^38^. The size, angle and number of side holes influence the penetration depth of these jets^41^. Adjusting these parameters could help in minimizing structures that considerably disrupt physiological flow conditions, reducing separation bubbles and mitigating risks of blood trauma.

In addition, the volume of stagnant blood in the whole system was computed as a percentage of total blood volume for the three configurations (Fig. 6). Stagnation volume decreased with increased flow rate, with the JF configuration showing the lowest stagnation volume of the three. In the FF configuration, stagnant blood made up less than 1% of the total blood volume at flow rates above 2 L/min, with stagnation primarily occurring in regions around the cannula where flow was restricted.

**Fig. 7:**
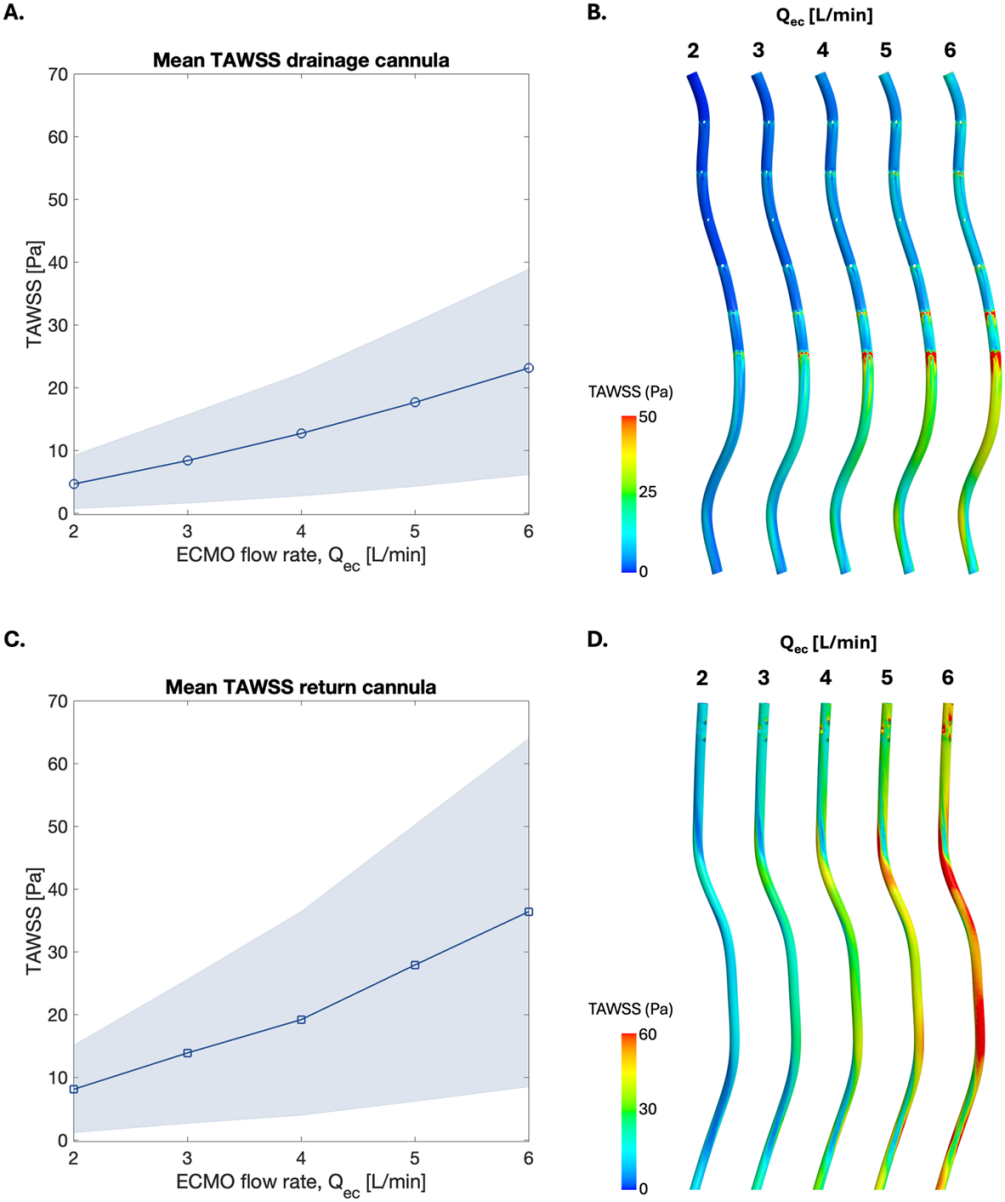
**A.** Mean time-averaged wall shear stress (TAWSS) at the inner wall of the drainage cannula. The shaded region indicates the 10th-90th percentile of TAWSS values. **B.** TAWSS distribution at the inner wall of the drainage cannula for different ECMO flow rates. **C.** Mean TAWSS at the inner wall of the return cannula. The shaded region indicates the 10th-90th percentile of TAWSS values. **D.** TAWSS distribution at the inner wall of the return cannula for different ECMO flow rates. Note that the scales differ in D compared to B.

### Mechanical stresses

Mean TAWSS at the inner wall of the cannulae increased with increased ECMO flow rate, exceeding physiological shear stress level of 12 Pa^18^ at flow rates above 3 L/min in the drainage cannula (Fig. 7A) and above 2 L/min in the return cannula (Fig. 7C). The 10th-90th percentile range also grew at higher flow rates, showing the high local variability of TAWSS in the cannula at high flow rates. In the drainage cannula, TAWSS was highest at the most proximal drainage holes for all flow rates, with a prolonged region of elevated TAWSS continuing downstream of the side holes (Fig. 7B). This pattern was especially prominent at the most proximal side holes, gradually decreasing towards the tip of the cannula. An exception to this was at hole row 3, where no signs of elevated TAWSS were seen due to the minimal drainage at this site. This was likely a result of cannulae positioning in relation to the anatomy of the IVC. TAWSS at the drainage cannula has been studied in the FJ configuration, both with a multistage and lighthouse drainage cannula^40^. As the wall shear stress inside the cannula is governed by the cannula flow, little difference is expected in the updated geometry. Similar WSS distributions were observed in the multistage drainage cannula in FF and FJ configurations.

In the return cannula, high TAWSS was observed both at the side holes and downstream of cannula bends stemming from the patient anatomy (Fig. 7D). While elevated WSS also developed downstream of the return cannula side holes, similar to the drainage cannula, these patterns were less pronounced. Cannula design choices are thus important for both drainage and return cannulae. For the return cannula, WSS distribution is also affected by the anatomy of the vascular system and impacted by insertion length. Larger regions depicted high WSS in the return cannula as compared to the drainage cannula, potentially increasing the risk of platelet activation and blood damage.

**Fig. 8:**
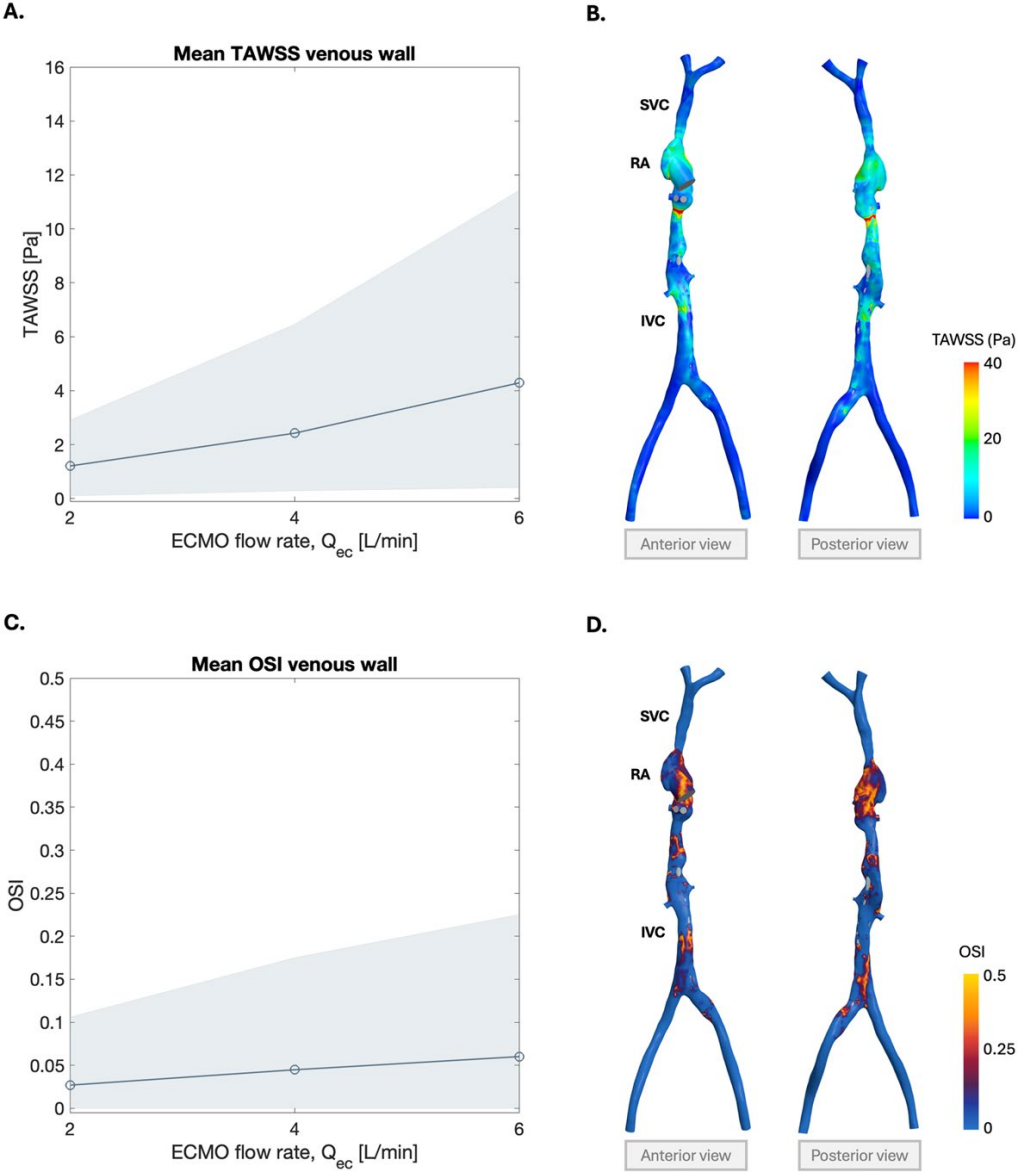
**A.** Mean time-averaged wall shear stress (TAWSS) at the vascular wall, including the right atrium (RA), inferior vena cava (IVC) and superior vena cava (SVC). The shaded region indicates the 10th-90th percentile of TAWSS values. **B.** TAWSS distribution at the vascular wall for ECMO flow rate 6 L/min. **C.** Mean oscillatory shear index (OSI) at the vascular wall. The shaded region indicates the 10th-90th percentile of OSI values. **D.** OSI distribution at the vascular wall for ECMO flow rate 6 L/min.

In addition to shear stresses on cannulae surfaces, the vascular wall itself is exposed to changes in hemodynamic conditions. Wall shear stress above 7 Pa has been considered elevated and associated with flow disturbances that may contribute to atherogenesis^42^. Moreover, vWf has been reported to unfold at lower stress levels in elongational flow than in shear flow, corresponding to levels as low as 7 Pa, which in turn may facilitate thrombus formation^22,23^. In this study, the mean TAWSS at the vascular wall was well below 7 Pa at all flow rates, reaching 4.3 Pa at 6 L/min (Fig. 8A). Beyond mean values, smaller regions well surpassed physiological shear stress as the flow rates increased, reaching 40 Pa just below the RA at 6 L/min (Fig. 8B).

Mean OSI at the wall remained low (0.06 at 6 L/min), indicating largely unidirectional flow (Fig. 8C). However, localized regions with high OSI values, approaching 0.5, were observed around the RA and in the vicinity of the drainage cannula side holes at 6 L/min flow rate (Fig. 8D). These localized regions of high OSI highlight areas of disturbed flow where oscillatory shear dominates, arising from the return cannula jet impinging into the RA and the drainage of blood through the side holes.

Regions of low TAWSS combined with high OSI have been linked to endothelial dysfunction, atherosclerosis and intimal thickening^16,42^. In this study, most of the vascular wall was exposed to physiological TAWSS and low OSI, suggesting that disturbed hemodynamics are restricted to local regions rather than systemic. This emphasizes the importance of both cannula positioning and flow settings, as small geometric or anatomical changes can create local areas of unfavorable wall shear stress conditions.

As TAWSS varied significantly along the inside of the cannula and at the vascular wall, it is worth noting that it may be misleading to solely rely on averaged values. Shear and elongational stresses are often simplified as one scalar, neglecting meaningful information on how the stresses deform or damage particles^43^. In prospective studies, stresses in the circuit and their coupling to blood trauma should be further investigated by studying shear and elongational stresses separately.

**Fig. 9:**
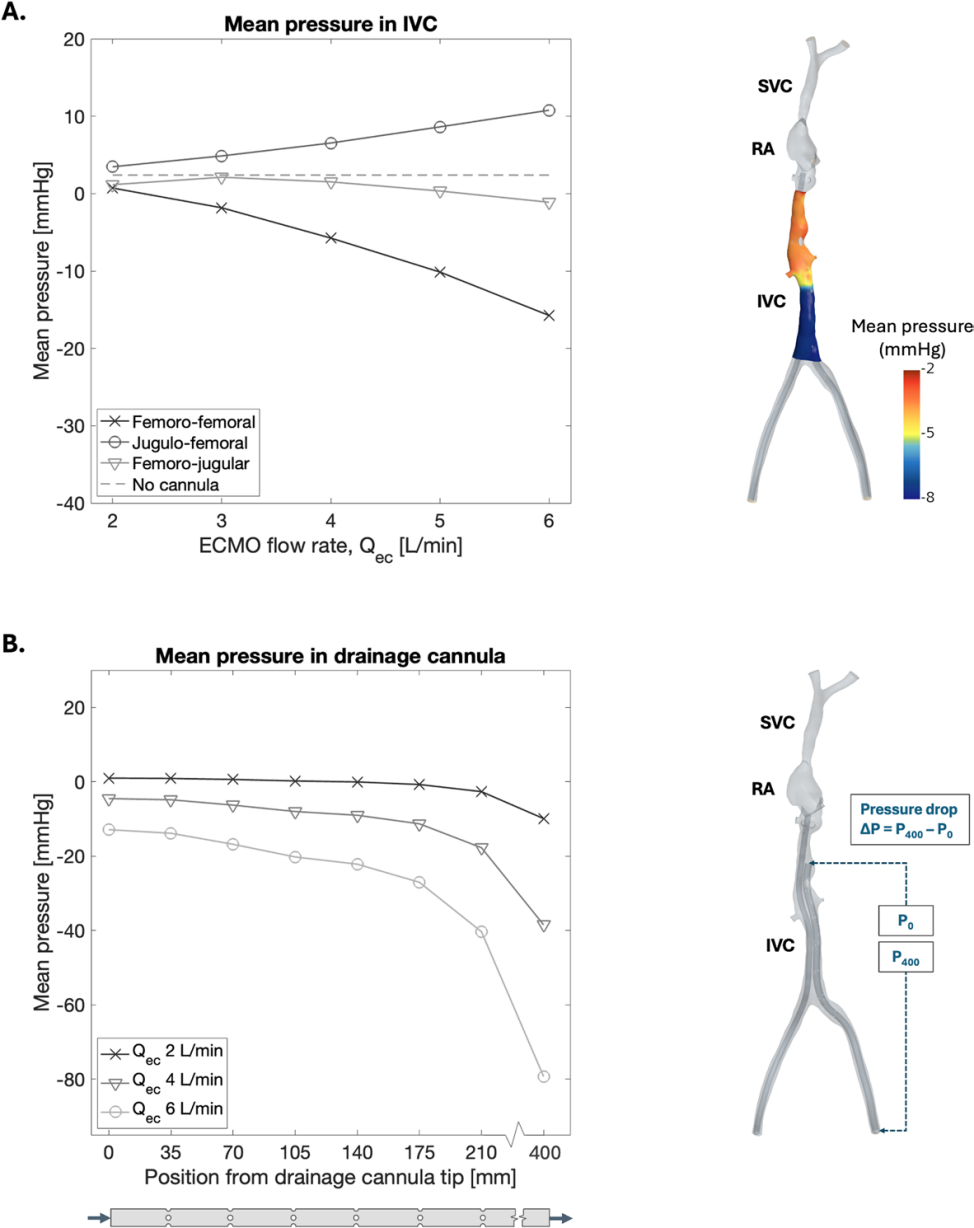
**A.** Mean pressure at the inferior vena cava (IVC) vessel wall at varying ECMO flow rates and for different cannula configurations. Pressure distribution in the IVC is shown to the right in femoro-femoral configuration with 4 L/min ECMO flow rate. **B.** Mean pressure along the drainage cannula in femoro-femoral (FF) configuration, from tip to the most distal location in the femoral vein, at varying ECMO flow rates.

### Pressure

Mean internal pressure at the vascular wall of the IVC decreased with increasing flow rate during FF cannulation, with positive values attained only at the lowest flow rate of 2 L/min (Fig. 9A). The pressure distribution in the IVC revealed an abrupt pressure decrease in the region below the hepatic veins, observed at all flow rates with FF cannulation. Comparatively, the mean vascular wall pressure stayed positive with FJ up to 5 L/min flow rate and with JF at all flow rates. Considerable negative pressures are associated with a risk of the vessel collapsing onto the cannula, causing cannula chattering^28^. In FF cannulation, especially during high ECMO flow rates, the negative pressures may thus indicate an elevated risk of vascular collapse and cannula chattering compared to JF and FJ cannulation.

The pressure difference between the inflow and outflow end of the cannulae increased with increasing flow rates. For the return cannula, this corresponded to pressure differences of 21.3 mmHg at 2 L/min, 51.1 mmHg at 4 L/min, and 85.8 mmHg at 6 L/min, while for the drainage cannula, corresponding values were 10.9 mmHg at 2 L/min, 34.0 mmHg at 4 L/min, and 66.4 mmHg at 6 L/min (Fig. 9B). As the model was cropped compared to reality, and the total drainage cannula length of 55 cm was not modelled, these pressure gradients are expected to be higher in clinical practice. Compared to measurements obtained by Broman et al. (2019)^44^, using whole blood at $37.5^{\circ }$C in a mock loop, the pressure gradient across the drainage cannula was approximately 13 mmHg at 2 L/min, 34 mmHg at 4 L/min, and 69 mmHg at 6 L/min. Note that these pressure gradients were measured from the most proximal side holes to the outflow end of the cannula closest to the ECMO pump.

## Limitations

The model was based on several simplifications and assumptions. A rigid walls assumption was incorporated while, in reality, veins are highly compliant and the RA changes in volume throughout the cardiac cycle. Adding compliance and vessel wall movement could theoretically bring the model closer to representing a real case, but the lack of information about the properties of the vessel wall introduces considerable challenges. Rather than improving the model, this approach is likely to introduce more uncertainty. In addition, constant inlet flow rates may affect the accuracy of the model, along with the assumption of a zero-pressure outlet at the tricuspid valve. Setting a condition at the tricuspid valve would over-constrain the boundary conditions due to the assumption of rigid walls and is therefore not feasible in the current model. Moreover, the inlet flow distribution in the different vessels were based on literature of the healthy individual. This may differentiate from the sick patient, where blood delivery can be redistributed to prioritize vital organs. Currently, details regarding the flow distribution at the inlets is lacking in literature, thus remaining unknown. Lastly, the patient-averaged geometry was derived from a small subset of healthy individuals, which may not accurately represent patients requiring ECMO. Anatomical variations in blood vessels, which are especially prominent in the venous system, may impact flow dynamics significantly.

Including the hepatic veins reduced the proportion of blood entering the model via the iliac veins and led to an increase in $R_f$ for the FJ configuration, which drains from the IVC (Supplemental Material, Figure S5). In addition, the presence of hepatic veins influenced the mean pressures in the IVC (Supplemental Material, Figure S6). These findings highlight the sensitivity to simplifications in the anatomical model, and the importance of consistency with model simplifications when performing comparisons.

## Conclusions

This work evaluated the impact of FF cannulation on native blood flow. Structures in the RA remained relatively unchanged by FF cannulation. Although FF has been associated with higher $R_f$ than FJ, this study showed a lower $R_f$ in FF compared to both FJ and JF. At increased flow rates, an increase in $R_f$ was observed. This was likely due to considerable drainage flow increase at the cannula tip and its proximity to the return cannula. Further, negative pressures developed in the IVC, potentially elevating the risk of vascular collapse in FF cannulation compared to JF or FJ. Non-physiological values of TAWSS were reached locally in the cannulae at flow rates $>2$ L/min. With increased flow, both mean and local TAWSS exceeded 12 Pa, increasing the risk of blood trauma. At the vascular wall, regions of elevated TAWSS and high OSI developed locally, particularly around the RA and drainage side holes, indicating disturbed flow and risk of endothelial dysfunction. These key factors highlight the importance of advancing knowledge on cannula flow dynamics to promote informed relevant clinical decisions.

## Supplementary Information

Supplementary Information.

## Data Availability

The datasets used and analysed in the current work are available from the corresponding author upon reasonable request.
