# Supplementary material for: Hemodynamics in femoro-femoral venovenous extracorporeal membrane oxygenation using large eddy simulations
Source: Sci Rep. 2025 Oct 9;15:35229. doi: 10.1038/s41598-025-22403-6 (PMC12511417; doi:10.1038/s41598-025-22403-6)
Supplement: Supplementary file 1 — Supplementary Information. [file 41598_2025_22403_MOESM1_ESM.pdf]

Supplemental material

Grid sensitivity

Grid convergence was performed on three meshes of 4.2M (coarse), 10.4M (mid) and 20.1M (fine) cells. Time averaged velocities, recirculation fraction, turbulent kinetic energy (TKE), wall shear stress (WSS), and pressure were computed for each along probe lines or in the right atrium (RA), see table 2. Velocities and RMS was plotted (Figure S1B - S1G) along probe lines located in the RA near the tip of the return cannula (Figure S1A), where velocity gradients were high. The two quantities that showed the most significant difference across meshes, namely recirculation fraction and pressure, were plotted for the three meshes (Figure S2). The selected mesh contained 10.4M cells, with the core cells being polyhedral cells and 6 prism layers. The grid was further refined at the cannulae holes, where velocity gradients increase.

Table S1: Mesh convergence for velocity, recirculation fraction, time averaged turbulent kinetic energy (TATKE), time averaged wall shear stress (TAWSS). For coarse meshes the difference to the fine mesh are shown.

		**4.2M**	**10.4M**	**20.1M**	
Quantity	Location	Diff. 20.1M [%]	Diff. 20.1M [%]	Value	Unit
Mean velocity	Probe line V	4.17	0.42	2.62	m/s
Mean velocity	Probe line H2	3.40	1.46	1.34	m/s
Mean velocity	Probe line H3	3.03	1.94	1.81	m/s
Recirculation fraction	Draining cannula outlet	1.65	0.53	30.56	%
TATKE	RA volume avg.	0.83	0.95	0.048	J/kg
TAWSS	RA surface avg.	1.98	1.34	10.24	Pa
Mean pressure	RA surface avg.	32.11	1.53	85.28	Pa

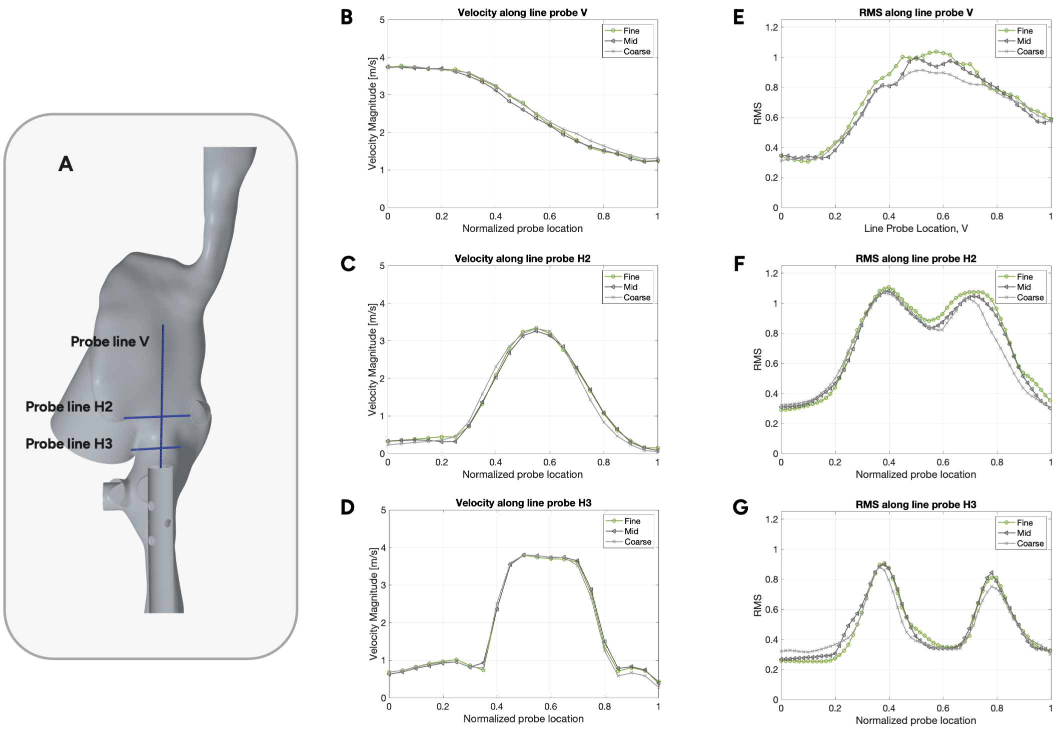

Figure S1: **A** Probe lines in right atrium. **B** - **D** Time averaged velocity along probe lines. **E** - **G** RMS along probe lines.

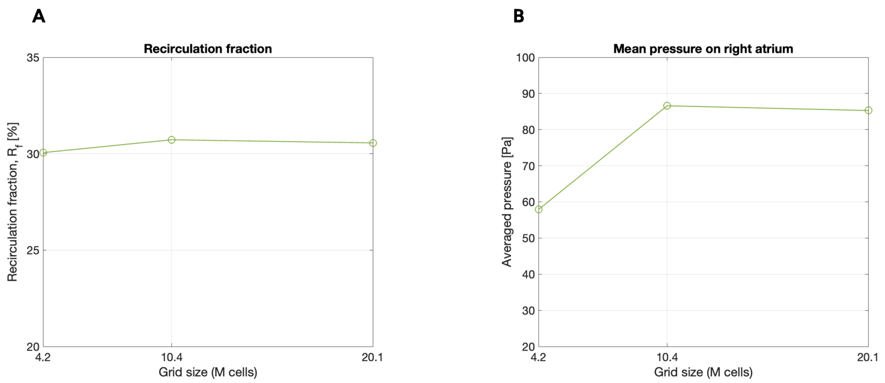

Figure S2: Grid sensitivity of **A** recirculation fraction and **B** mean pressure.

Time sensitivity

To ensure the flow was fully developed at 3 s for all simulations, simulations were run for 4 s of physical time for the case with lowest ECMO flow rate. The flow field was averaged over the last second for three different cases, namely a 2 s, 3 s and 4 s simulation. The oxygenated blood in the RA with the line integral convolution following the averaged velocity of the fluid is shown in Figure S3. The large rotating structure at the center of the RA was fully developed at 2 s, however the smaller rotating structure at the auricle needed additional time to fully develop. At 3 s, there was no significant difference in the flow structure to that of 4 s. Further, the oxygenated blood was considered fully dissipated at 3 s.

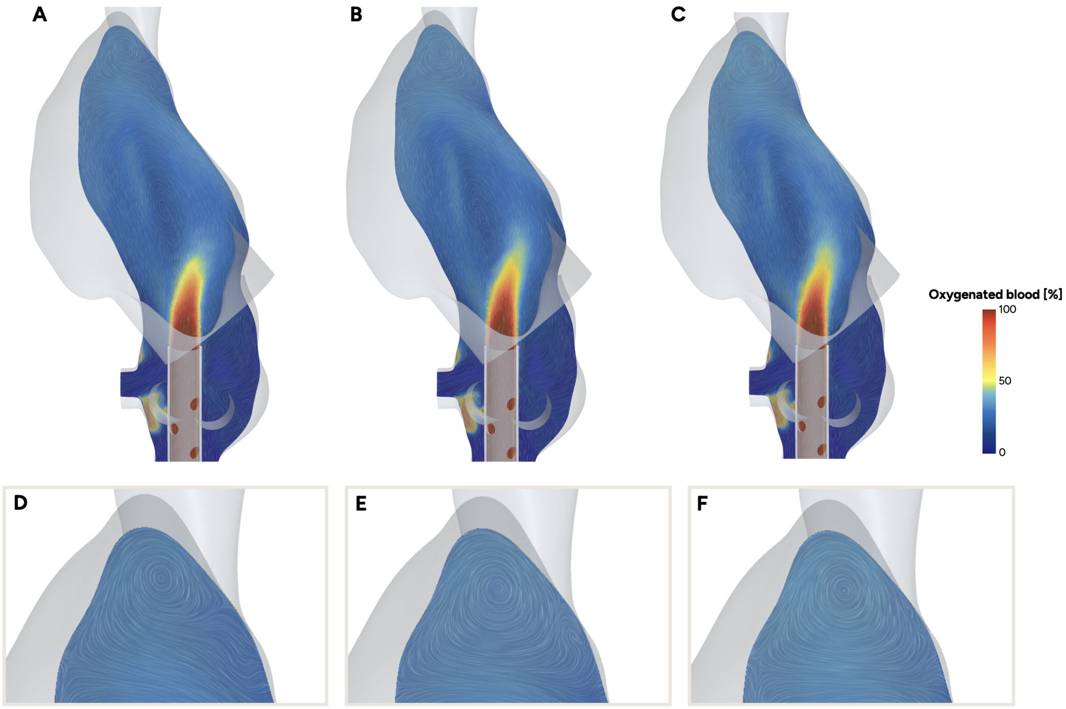

Figure S3: Oxygenated blood in the right atrium with the line integral convolution following the velocity of the fluid. **A** Flow field time-averaged over 1-2 s. **B** Flow field time-averaged over 2-3 s. **C** Flow field time-averaged over 3-4 s. **D-F** Closer look at the flow in the right atrium auricle for cases **A-C**.

Cannula orientation

The drainage cannula was rotated 45° to investigate sensitivity to cannula orientation due to the proximity to the vessel walls during FF cannulation. Rf showed no noticeable difference when comparing the two cases (Figure S4A). Drainage fraction displayed a small difference at the tip and first set of side holes (Figure S4B), likely due to partial occlusion of side holes with the neutral cannula.

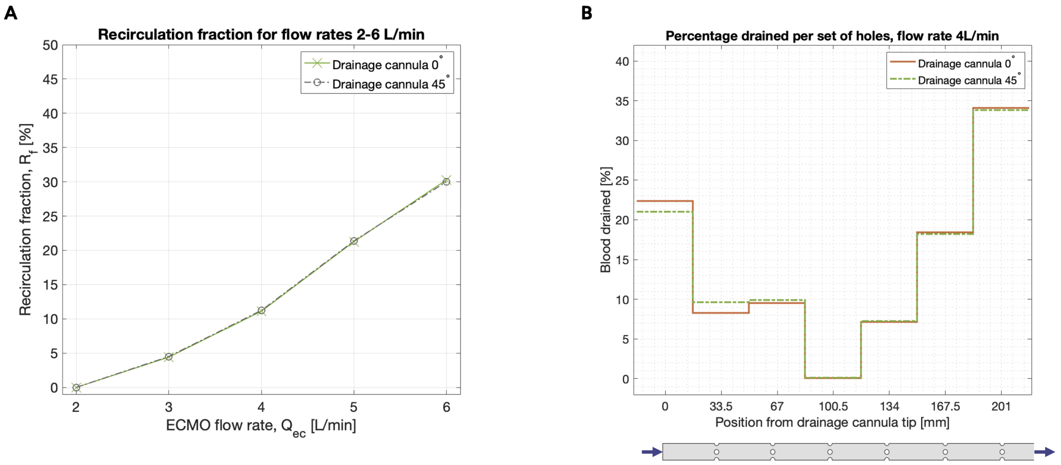

Figure S4: Sensitivity to drainage cannula direction. **A.** Recirculation fraction, R_f, for neutral drainage cannula (0°) compared to when rotated 45°. **B.** Drainage fraction throughout cannula for neutral drainage cannula (0°) compared to when rotated 45°.

Sensitivity to disregarding hepatic veins

Incorporating hepatic veins resulted in a slight decrease in R_f for JF (mean decrease 1.2 ± 0.8%) but a large increase in R_f for FJ (mean increase 8.6 ± 5.0%). With blood entering the model through the hepatic veins, the proportion of blood entering via the iliac veins was reduced. Consequently, in the FJ configuration, where blood is drained through the IVC, a larger volume of blood was drained near the tip of the cannula, in proximity to the oxygenated blood from the return cannula. This led to an increase in R_f. In the JF configuration, blood is drained through the SVC, making R_f less sensitive to the inclusion of the hepatic veins compared to FJ.

The mean pressure in the IVC decreased slightly after incorporating the hepatic veins (Figure S6), with the largest difference observed for the FJ configuration at an ECMO flow rate of 6 L/min. In the updated model, the mean pressure shows a consistent decrease with increasing flow rate, an effect not seen in the previous model.

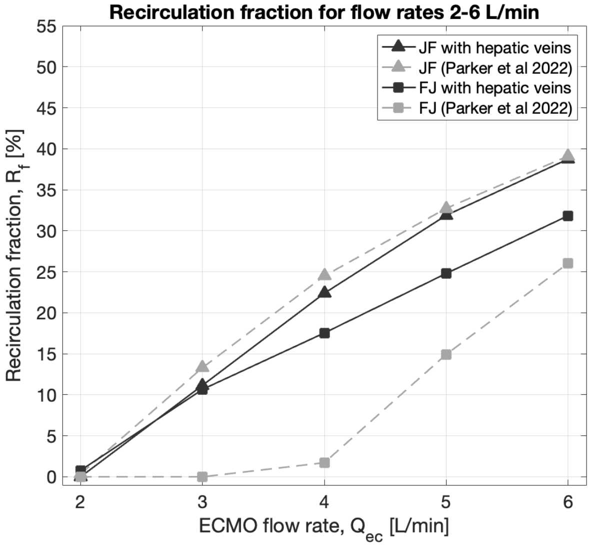

Figure S5: Recirculation fraction, R_f, under jugulo-femoral (JF) and femoro-jugular (FJ) configurations without the presence of hepatic veins (Parker et al. 2022a), and with hepatic veins incorporated into the model.

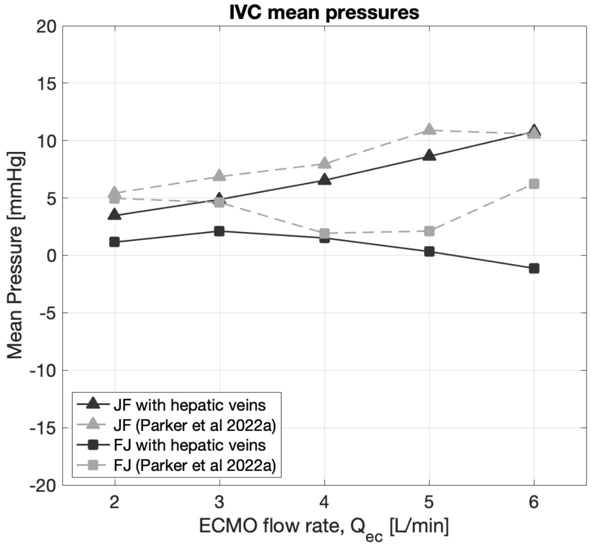

Figure S6: Mean pressure in the inferior vena cava (IVC), under jugulo-femoral (JF) and femoro-jugular (FJ) configurations without the presence of hepatic veins (Parker et al. 2022a)), and with hepatic veins incorporated into the model.

Comparison with clinical data

Fisser et al. (2022) measured the effective ECMO flow (Q_{EFF}) in FJ and JF configurations using ultrasound dilution technology, defining Q_{EFF} as $Q_{EFF} = Q_{EC} \times (1 - R_f)$. A quadratic regression line was fitted to the mean scatter plot values, with 95% confidence intervals calculated for each configuration. Figure S7 compares Q_{EFF} from clinical data by Fisser et al. with results from the FJ and JF simulations conducted in this study. The simulated data fell within the 95% confidence intervals of the clinical data for both configurations.

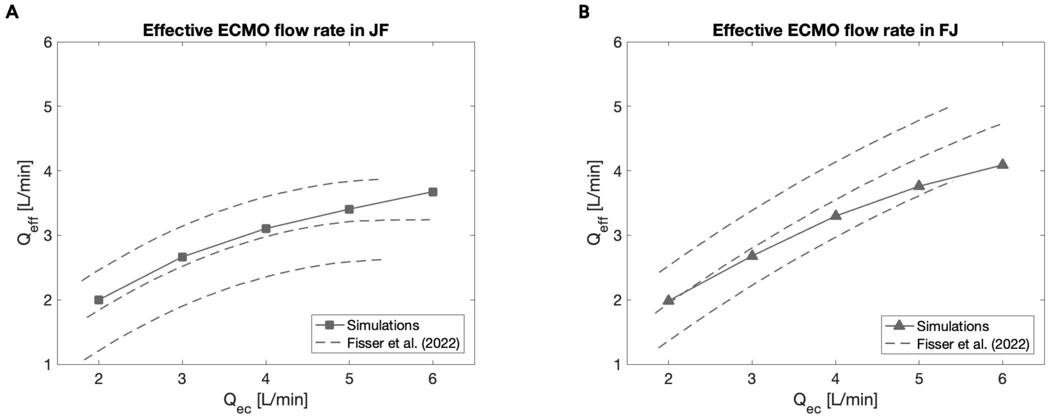

Figure S7: Comparison in effective ECMO flow rate (Q_{EFF}) to clinical data by Fisser et al. (2022), shown as mean and 95% confidence intervals, for configurations **A.** jugulo-femoral (JF) **B.** femoro-jugular (FJ).
